# Pregnant Women’s Experiences of Obstetric Abdominal Ultrasound: A Qualitative Study Exploring Emotional, Cognitive, and Relational Dimensions

**DOI:** 10.3390/healthcare14203472

**Published:** 2026-10-16

**Authors:** Ayse Sezer Balci, Cigdem Can Bayrak, Meliha Akpınar Alaoglu

**Affiliations:** 1 Department of Public Health Nursing, Faculty of Health Sciences, Burdur Mehmet Akif Ersoy University, Burdur 15030, Türkiye; asbalci@mehmetakif.edu.tr; 2 Department of Obstetrics and Gynecology, Bucak State Hospital, Burdur 15300, Türkiye; 3 Geriatric Care Department, Istanbul Atlas University, Istanbul 34408, Türkiye

**Keywords:** obstetric ultrasound, pregnancy, qualitative research, maternal identity, woman-centered care

## Abstract

**Aim:** This study aimed to explore pregnant women’s experiences of obstetric abdominal ultrasound and examine the emotional, cognitive, and relational dimensions of these experiences. **Methods:** A qualitative descriptive study was conducted with 20 pregnant women attending routine obstetric ultrasound examinations in Türkiye. Data were collected through semi-structured interviews and analyzed using Braun and Clarke’s reflexive thematic analysis. **Results:** Five themes were identified, reflecting the emotional, cognitive, relational, and embodied dimensions of women’s ultrasound experiences. Women’s experiences of obstetric ultrasound were characterized by emotional ambivalence, the transformation of pregnancy into a tangible and relational reality through fetal visualization, participants’ evolving sense of maternal identity, reassurance associated with supportive communication, and perceptions of preparedness for childbirth and motherhood. **Conclusions:** The findings highlight the importance of integrating woman-centered communication and emotional support into routine obstetric ultrasound to address women’s informational, emotional, and relational needs during antenatal care.

## 1. Introduction

Obstetric abdominal ultrasound has become an indispensable component of contemporary antenatal care, providing essential information on fetal growth, anatomical development, placental location, gestational age, and fetal well-being throughout pregnancy [1]. However, obstetric ultrasound is increasingly recognized as more than a biomedical imaging technique. Emerging evidence suggests that it also constitutes a psychosocial, emotional, and relational experience that shapes women’s perceptions of the fetus, pregnancy, and emerging motherhood [2]. Reflecting this broader perspective, the World Health Organization recommends at least one ultrasound examination before 24 weeks of gestation to support a positive pregnancy experience, emphasizing not only clinical outcomes but also women’s experiences of care during pregnancy [3].

In many healthcare systems, obstetric ultrasound is routinely presented as a reassuring procedure, and women commonly attend these examinations expecting confirmation of fetal health and reassurance that the pregnancy is progressing normally [4]. Consequently, ultrasound may evoke feelings of relief, happiness, and security. A recent systematic review demonstrated that antenatal imaging could promote prenatal attachment and positively influence parental experiences. However, these effects appear to vary with factors such as imaging context, communication with healthcare professionals, the information provided, and the presence of abnormal findings [5]. Similarly, previous studies have shown inconsistent findings regarding the effects of obstetric ultrasound on maternal anxiety and maternal–fetal attachment, suggesting that the ultrasound experience encompasses complex psychosocial dimensions [6,7].

Recent evidence further indicates that fetal visualization enables women to perceive the fetus as a more concrete and relational entity and may facilitate maternal–fetal attachment and adaptation to pregnancy [5,8]. Consequently, obstetric ultrasound can be understood not merely as an imaging modality but also as an experience that shapes the psychological and relational meaning of pregnancy.

The meaning attributed to ultrasound extends beyond the imaging procedure itself and is influenced by women’s prior experiences, risk perceptions, and interactions with healthcare professionals. Existing evidence suggests that the psychosocial impact of prenatal ultrasound cannot be fully explained by quantitative outcomes alone and that communication plays a pivotal role in shaping women’s emotional experiences [1,9]. Inadequate communication and uncertainty have been reported to increase anxiety and emotional vulnerability during pregnancy [1,10]. Therefore, obstetric ultrasound may be conceptualized as a care encounter with substantial psychosocial significance rather than merely a technical assessment.

Nevertheless, previous studies have predominantly focused on diagnostic outcomes and isolated constructs such as anxiety, depressive symptoms, or prenatal attachment, providing limited insight into how women experience routine obstetric abdominal ultrasound and how its emotional, cognitive, and relational dimensions intersect within their lived experiences [11,12]. Consequently, qualitative studies examining the multidimensional psychosocial meaning of routine obstetric ultrasound remain scarce.

Understanding women’s experiences of obstetric ultrasound is essential for advancing woman-centered prenatal care, strengthening therapeutic communication, and supporting maternal psychological well-being. This study aimed to explore pregnant women’s experiences of obstetric abdominal ultrasound and examine the emotional, cognitive, and relational dimensions of these experiences. The study was informed by a woman-centered care perspective and by the assumption that pregnancy experiences are socially and relationally constructed.

## 2. Methodology

### 2.1. Study Design

This qualitative descriptive study was informed by a constructivist perspective and a woman-centered care perspective. A qualitative descriptive design was selected because the study aimed to provide a detailed and clinically relevant account of pregnant women’s experiences of routine obstetric abdominal ultrasound while remaining closely grounded in participants’ own descriptions and meanings.

From a constructivist perspective, participants’ experiences were understood as situated and actively constructed through their individual histories, previous obstetric experiences, expectations, interactions with healthcare professionals, and the sociocultural context of pregnancy. Accordingly, the study did not assume that ultrasound had a single or predetermined meaning. Instead, meanings attributed to the ultrasound experience were explored through participants’ accounts and interpreted in relation to their personal and relational contexts.

The woman-centered care perspective guided attention to dimensions of the ultrasound encounter that extend beyond technical and diagnostic aspects, including emotional safety, communication, privacy, bodily autonomy, and women’s involvement in understanding and interpreting their pregnancy experiences. This perspective informed the interview focus and the interpretation of findings rather than functioning as a predefined coding framework.

Reflexive thematic analysis, as described by Braun and Clarke, was used to analyze the interview data [13]. This approach was considered compatible with the qualitative descriptive design because it enabled the identification of patterns of meaning across participants’ accounts while retaining sufficient interpretive depth to examine how women constructed meanings around ultrasound, pregnancy, and emerging motherhood. The analysis was primarily inductive and focused predominantly on the semantic meanings expressed by participants, while interpretive attention was also given to the relational and sociocultural meanings underlying these accounts. The study was reported in accordance with the Consolidated Criteria for Reporting Qualitative Research (COREQ), a 32-item checklist for qualitative interviews and focus groups [14]. The completed COREQ checklist is provided as Supplementary File S1.

### 2.2. Research Team and Reflexivity

The transcripts were coded by two members of the research team (A.S.B. and M.A.A.) using Word and Excel. The researchers worked collaboratively throughout the coding process, discussing initial codes, developing candidate themes, and refining interpretations of patterns across the dataset. Rather than seeking inter-coder reliability or a single objective coding agreement, they engaged in reflexive discussion to consider different interpretations and develop a coherent account of participants’ experiences.

The interviews were conducted by a female obstetrician (C.C.B.) with experience in women’s health and formal training in qualitative interviewing methods. She also performed the routine obstetric ultrasound examinations immediately preceding the interviews. Participants were approached by the same researcher after completing their ultrasound examination and were invited to participate in the study. Written informed consent was obtained before the interview. Participants were explicitly informed that participation was voluntary, that they could decline participation or withdraw from the interview at any time, and that their decision would not affect their medical care or relationship with the healthcare service.

The interviewer’s dual role as both clinician and researcher was recognized as a potential source of power asymmetry and social desirability bias. Participants may have been reluctant to express negative views about the ultrasound examination or the communication they had received from the clinician who had just provided their care. To reduce perceived pressure, participation was presented as entirely voluntary, refusal or withdrawal was explicitly stated to have no consequences for clinical care, and interviews were conducted in a private room with no other individuals present.

The researchers maintained reflexive field notes throughout data collection and analysis to document their assumptions, reactions, and potential influence on the interpretation of participants’ accounts. During the analysis, A.S.B. and M.A.A. reflexively discussed the developing codes and themes, including the possibility that positive accounts of reassurance and communication could partly reflect the clinical relationship between participants and the interviewer. The analysis, therefore, attended to accounts of anxiety, uncertainty, privacy concerns, and vulnerability alongside positive experiences. The interviewer’s clinical position and its potential influence on data generation and interpretation were considered throughout the analytic process.

### 2.3. Setting and Participants

The study was conducted in the obstetric outpatient clinic of a tertiary hospital in Türkiye from late April to June 2026. Participants were recruited using maximum-variation purposive sampling to capture variation in age, gestational age, parity, obstetric history, and previous pregnancy experiences. This approach was used to include women with differing obstetric and experiential backgrounds rather than to achieve statistical representativeness.

Of the 46 women approached, 17 declined participation, and 9 withdrew before completing the interview. The final analytic sample comprised 20 women, including 6 in the second trimester and 14 in the third trimester. Women were eligible if they were aged 18 years or older, had undergone a routine obstetric abdominal ultrasound, were able to communicate in Turkish, and agreed to participate voluntarily. Women with severe psychiatric disorders or communication difficulties that could interfere with the interview process were excluded. The study had a focused research aim concerning women’s experiences of routine obstetric abdominal ultrasound, and participants had direct experience with the phenomenon under investigation. The interviews were individual and in-depth, lasting approximately 30–45 min. Data collection and preliminary analysis were conducted concurrently, allowing the researchers to assess the contribution of subsequent interviews to the developing thematic structure. During the later interviews, participants’ accounts continued to elaborate and provide further variation within the developing themes. Sample adequacy was assessed iteratively during data collection and analysis based on the concept of information power. The focused research aim, the specificity of the participant group, direct experience with the phenomenon, and the richness of the individual interviews supported sufficient information power with 20 participants [15]. All participants had low-risk pregnancies without additional medical complications and underwent routine obstetric abdominal ultrasound as part of their antenatal follow-up. The examinations were performed using a Mindray DC-T6 ultrasound system (Shenzhen Mindray Bio-Medical Electronics Co., Ltd., Shenzhen, China) with two-dimensional (2D) B-mode imaging and lasted approximately 5 min. No companions were present during the examinations or interviews. Information regarding participants’ previous ultrasound exposure was not systematically collected and, therefore, was not included in the analysis. No abnormal or suspicious fetal findings were identified during the ultrasound examinations.

### 2.4. Data Collection

Data were collected through individual semi-structured, in-depth interviews. The interview guide was developed based on the relevant literature and the study aims and was pilot-tested with two pregnant women to assess the clarity and comprehensibility of the questions. The pilot interviews were not included in the final analysis.

The interviews explored women’s emotional responses to ultrasound examinations, perceptions of the fetus, experiences of communication with healthcare professionals, and experiences of privacy during the examination. Interviews were conducted immediately after the routine ultrasound examination performed by the interviewer. No individuals other than the participant or the interviewer were present during the interviews.

With written informed consent, all interviews were audio-recorded, and the researcher documented field notes. Interviews lasted approximately 30–45 min. Data collection and analysis were conducted concurrently, allowing emerging concepts to be explored in subsequent interviews [15].

### 2.5. Ethical Considerations

The study was approved by the Non-Interventional Clinical Research Ethics Committee of Burdur Mehmet Akif Ersoy University, Türkiye (Approval No. GO 2026/2925). Written informed consent was obtained from all participants prior to data collection, and the study was conducted in accordance with the principles of the Declaration of Helsinki. Given that some participants reported previous adverse obstetric experiences, including pregnancy loss and stillbirth, particular attention was given to participants’ emotional comfort during the interviews. Before the interview, participants were informed that participation was voluntary and that they could decline to answer any question, pause or terminate the interview, or withdraw from the study at any time without affecting their medical care. Interviews were conducted in a private room after the ultrasound examination, with no other individuals present. The interviewer monitored participants’ emotional responses throughout the interview and avoided pressuring participants to discuss experiences they did not wish to disclose.

### 2.6. Data Analysis

All interviews were audio-recorded and transcribed verbatim by the researchers. Data were analyzed using Braun and Clarke’s six-phase reflexive thematic analysis approach. The analysis was primarily inductive and semantic, with interpretive attention to relational and sociocultural meanings. First, the transcripts were read repeatedly to achieve familiarity with the data and to gain an overall understanding of participants’ experiences. Second, initial codes were generated inductively across the dataset by A.S.B. and M.A.A. using Microsoft Word and Microsoft Excel (Microsoft Corporation, Redmond, WA, USA). Third, related codes were examined and organized into potential themes and subthemes. Fourth, the candidate themes were reviewed and refined through collaborative discussions between the researchers, considering their coherence, distinctiveness, and relationship to the coded data and the dataset. Fifth, the themes were defined and named, and their boundaries and relationships were further clarified through reflexive discussion. Finally, the themes were organized into an interpretive account of pregnant women’s experiences of obstetric ultrasound.

Throughout the analytical process, the researchers engaged in reflexive discussions to consider alternative interpretations and the influence of their clinical and professional perspectives on the analysis. The aim was not to achieve inter-coder reliability or consensus as indicators of analytic validity, but to develop a rich and coherent interpretation of the dataset through collaborative, reflexive engagement. Data collection and analysis were conducted concurrently, allowing emerging concepts to be explored in subsequent interviews. Data collection ceased when the researchers judged that sufficient information power had been achieved for the focused research question and analytic approach.

The categorical variables presented in Table 1 were not derived retrospectively from the thematic coding process. Rather, they were obtained systematically through direct questions included in the semi-structured interview guide and recorded during the interviews. These responses were subsequently summarized descriptively as frequencies and percentages. They were used to characterize the sample and contextualize the qualitative findings rather than as quantitative outcome measures.

#### 2.6.1. Development of the Conceptual Model

**Figure 1:**
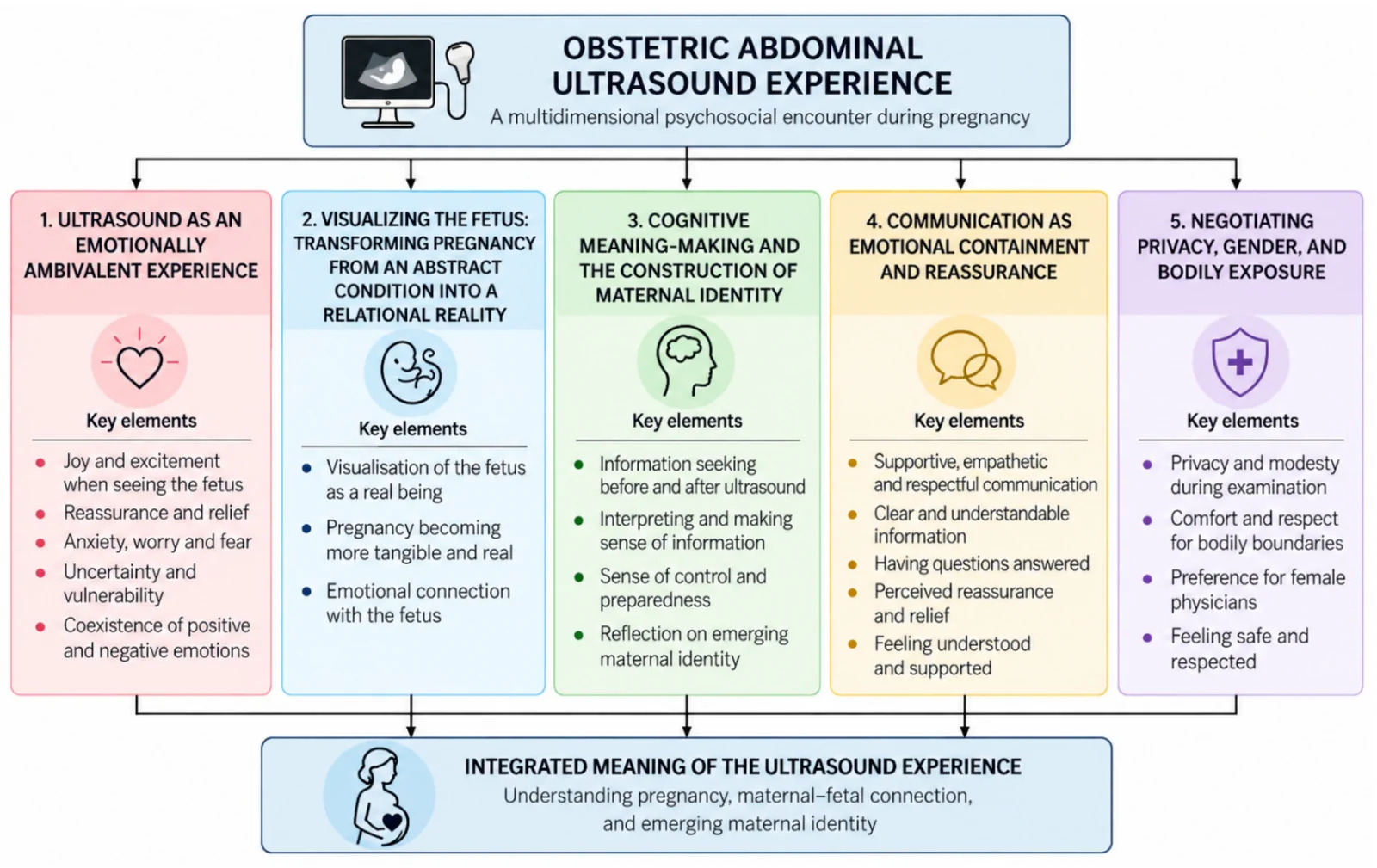
The interpretive conceptual model illustrates the interconnected dimensions of pregnant women’s experiences of obstetric abdominal ultrasound.

The conceptual model presented in Figure 1 was developed as an interpretive synthesis of the five themes generated through the reflexive thematic analysis. The model was developed after the initial codes and candidate themes had been reviewed and refined and was not used as a predefined framework for coding or theme generation. Its purpose was to visually represent the interconnected dimensions of participants’ experiences rather than to propose a causal model.

The five themes were conceptualized as interconnected rather than independent domains. Emotional experiences reflected the coexistence of excitement, reassurance, anxiety, and uncertainty; cognitive meaning-making captured how women interpreted fetal development and their changing pregnancy experiences; relational experiences reflected the meaning attributed to fetal visualization and connection; communication and reassurance represented the interpersonal processes through which information and emotional support were experienced; and privacy, gender, and bodily exposure represented the embodied and sociocultural dimensions of the ultrasound encounter. These dimensions were brought together under the central concept of integrated meaning of the ultrasound experience, reflecting how women interpreted pregnancy, developed a relationship with the fetus, and negotiated their emerging maternal identities.

The model was informed by the study’s constructivist and woman-centered perspectives but was not derived from a predefined theoretical model. It therefore represents an interpretive synthesis grounded in participants’ accounts and the analytic development of the themes.

#### 2.6.2. Trustworthiness

The trustworthiness of the study was established according to Lincoln and Guba’s criteria of credibility, transferability, dependability, and confirmability [16]. Credibility was enhanced through concurrent data collection and analysis, the inclusion of verbatim quotations, and regular analytical discussions within the research team. Transferability was facilitated by providing a rich description of the study setting, participants, and data collection procedures. Dependability was ensured through detailed documentation of the research process and maintenance of an audit trail. Confirmability was supported by reflexive field notes and the preservation of analytical memos and decision trails throughout the study.

## 3. Results

A total of 20 pregnant women participated in the study. Most participants were aged between 30 and 33 years, were in the third trimester of pregnancy, and several were experiencing their first pregnancy. Some women had previous adverse obstetric experiences, including cesarean birth, miscarriage, ectopic pregnancy, or stillbirth, whereas others reported chronic health conditions such as gestational diabetes, hypothyroidism, asthma, migraine, and coagulation disorders (Table 1).

Across participants, routine obstetric abdominal ultrasound was experienced as a multidimensional psychosocial encounter encompassing emotional, cognitive, relational, and bodily dimensions. Five interrelated themes emerged from the analysis: (1) ultrasound as an emotionally ambivalent experience; (2) visualizing the fetus: transforming pregnancy from an abstract condition into a relational reality; (3) cognitive meaning-making and the construction of maternal identity; (4) communication as emotional containment and reassurance; and (5) negotiating privacy, gender, and bodily exposure (Figure 1).

### 3.1. Theme 1. Ultrasound as an Emotionally Ambivalent Experience

Participants described obstetric ultrasound as an emotionally ambivalent experience in which excitement and reassurance coexisted with fear, anxiety, and uncertainty. For most women, the examination was not a neutral or routine clinical assessment; rather, it was a moment when hopes for fetal well-being were temporarily brought into contact with the possibility of an unexpected finding.

Excitement was commonly associated with the opportunity to see the fetus and observe its growth. Participants described each examination as meaningful because fetal development could be visually experienced over time. As P5 explained, “*I am excited because I will see my baby. I wonder how much the baby has grown*.” Similarly, P20 stated, “*Every visit feels special to me because the baby is growing more each time*.” These accounts suggest that the emotional significance of ultrasound was partly derived from the anticipation of encountering a visibly developing fetus rather than from the diagnostic function of the examination alone.

At the same time, anticipation was accompanied by uncertainty about what the examination might reveal. P4 described this coexistence directly: “*It is a mixture of everything—excitement, but also anxiety about whether there might be a problem or a developmental issue*.” For women with previous adverse obstetric experiences, this uncertainty could become particularly salient. For example, a participant with a previous stillbirth described persistent anticipatory anxiety: “*I came feeling nervous because of my previous stillbirth. No matter what, there is always fear and anxiety*” (P1). This experience contrasted with accounts in which ultrasound-related uncertainty was present but less distressing, suggesting that previous obstetric experiences may shape the emotional intensity attributed to the examination. For some women, the examination itself created a heightened sense of vulnerability because the possibility of an unfavorable finding lingered until it was completed. This was particularly evident among women with previous adverse obstetric experiences. Thus, reassurance was not experienced as the absence of anxiety; rather, reassurance and anxiety were often intertwined within the same encounter.

These accounts indicate that reassurance and vulnerability were not necessarily opposing experiences. Rather, reassurance and anxiety appeared to coexist within the same ultrasound encounter. Women hoped that the examination would confirm fetal well-being, yet the possibility of an unexpected finding remained present throughout the experience. Thus, the emotional meaning of ultrasound appeared to be shaped by an ongoing tension between hope for reassurance and awareness of uncertainty.

### 3.2. Theme 2. Visualizing the Fetus: Transforming Pregnancy from an Abstract Condition into a Relational Reality

Participants described ultrasound as an encounter that transformed pregnancy from an abstract or partially invisible experience into a more tangible and relational reality. Seeing the fetus on the screen appeared to give physical visibility to an experience that could otherwise remain difficult to fully comprehend, particularly in relation to fetal growth and the emerging sense of becoming a mother.

The image appeared to provide a concrete reference through which the participant could make sense of her changing identity and relationship to the pregnancy. Similarly, one participant emphasized the emotional significance of repeatedly seeing the fetus: “*I am excited because I will see my baby. That is why every appointment feels special*” (P18).

Rather than describing ultrasound solely as a diagnostic procedure, participants therefore attributed relational meaning to the visual encounter. Seeing the fetus appeared to make pregnancy more perceptible and emotionally immediate, allowing participants to connect bodily changes and fetal development with an emerging sense of the baby as a relational presence.

Among primigravid participants, ultrasound was sometimes described as particularly novel and emotionally salient. A 28-year-old primigravid participant explained, “*Because this is my first pregnancy, everything is very new and exciting. There are a little anxiety and excitement at the same time*” (P7). This account illustrates that, for some women experiencing pregnancy for the first time, ultrasound was embedded within the broader process of adapting to an unfamiliar pregnancy experience rather than being experienced solely as a diagnostic procedure.

Taken together, these accounts indicate that the meaning of ultrasound was not limited to obtaining medical information. For some participants, visualizing the fetus functioned as a way of making pregnancy more concrete and connecting fetal development with their developing sense of motherhood. The image provided a tangible point of reference through which an otherwise largely internal and bodily experience could be interpreted and shared, linking bodily sensations, fetal development, and the emerging maternal role within a single experiential frame.

Sharing the ultrasound experience with close others was also described as part of its emotional meaning. A 28-year-old primigravid participant reported, “*First I tell my husband. Then I give the good news to my mother and close friends*” (P7). For some participants, sharing appeared to extend the emotional experience of ultrasound beyond the clinical encounter and into close interpersonal relationships.

Overall, the visual dimension of ultrasound enabled women to perceive the fetus not merely as a biological entity but as an increasingly distinct relational presence, thereby deepening the emotional meaning of pregnancy.

### 3.3. Theme 3. Cognitive Meaning-Making and the Construction of Maternal Identity

Participants described ultrasound as an opportunity to make sense of pregnancy by connecting visual information about fetal development with their experiences of becoming a mother. The examination was therefore experienced not only as a source of medical information but also as a reference point through which participants interpreted bodily changes, fetal development, and their changing relationship with pregnancy.

One participant explained, “I feel more like a mother now. I did not fully understand this feeling during my first pregnancy, but now I feel it much more” (P2).

This account illustrates how the ultrasound image could become incorporated into participants’ personal meaning-making. Rather than simply confirming fetal development, the visual encounter appeared to provide a tangible reference through which the participant interpreted her changing experience of motherhood.

Participants also described using ultrasound findings to interpret the progression of pregnancy. Information about fetal growth and development helped them connect what they saw on the screen to the physical and emotional changes they were experiencing during pregnancy.

A 26-year-old primigravida at 19 weeks stated that she felt “closer” to the baby after the ultrasound and described her maternal feelings as positive (P16). This account suggests that fetal visualization could become incorporated into women’s ongoing interpretation of pregnancy and their transition toward motherhood, although the intensity and meaning of this experience varied across participants.

Overall, participants described meaning-making as an ongoing process through which they integrated visual information, bodily experiences, and emotional responses to pregnancy. The ultrasound encounter appeared to provide a concrete reference for interpreting these experiences, while the developing sense of motherhood emerged as part of this broader process rather than as a direct outcome of the examination.

### 3.4. Theme 4. Communication as Emotional Containment and Reassurance

Communication with healthcare professionals emerged as one of the most influential factors shaping women’s ultrasound experiences. Participants emphasized that the emotional impact of ultrasound depended not only on the examination itself but also on how information was communicated, how questions were addressed, and how healthcare professionals responded to their emotional needs. Women reported feeling calmer, safer, and more in control when clinicians communicated in an explanatory, supportive, and empathetic manner:

*“You explain things in a way that reassures me and helps me understand”.*

*(P14)*

*“I feel more comfortable here because there is eye contact and communication”.*

*(P13)*

For many participants, communication served as a source of emotional support that reduced anxiety and fostered a sense of security. Clear and understandable explanations alleviated fears regarding fetal health and diminished feelings of uncertainty. Several women also noted that trust in healthcare professionals reduced feelings of embarrassment and made them more willing to accept intimate procedures, such as vaginal examinations, when medically indicated.

Overall, communication functioned as more than an exchange of information; it represented a relational process through which emotional vulnerability was acknowledged, anxiety was contained, and trust was strengthened.

### 3.5. Theme 5. Negotiating Privacy, Gender, and Bodily Exposure

Participants described ultrasound examinations as embodied experiences involving privacy, bodily exposure, and vulnerability. Although most women regarded ultrasound as a medically necessary procedure, they emphasized the importance of respectful communication, privacy, and sensitivity to gender during the examination.

A recurring finding was participants’ preference for female physicians during obstetric examinations. Women frequently reported feeling more comfortable exposing their bodies to female healthcare professionals and experiencing less embarrassment during examinations:

“I feel more comfortable when the doctor is a woman. With a male doctor, you feel more exposed”.

(P1)

“I generally prefer female doctors because I think they understand me better”.

(P18)

Participants also emphasized the importance of environmental conditions that protected privacy, including limiting the number of people present, providing physical barriers such as curtains, and ensuring respectful practices regarding bodily exposure. Attention to privacy was described as reducing embarrassment and increasing trust, whereas inadequate privacy or the presence of unfamiliar individuals could heighten feelings of vulnerability and emotional tension.

Not all participants experienced the physical aspects of ultrasound in the same way. One participant described mild discomfort during the examination, noting, “When you press a little during contractions, it hurts a little” (P15).

This contrasting account highlights that a generally reassuring or emotionally meaningful ultrasound experience did not necessarily imply complete physical comfort. The acceptability of the examination appeared to depend not only on the medical purpose of ultrasound but also on how women experienced privacy, bodily boundaries, and interpersonal interactions during the encounter.

## 4. Discussion

This study explored pregnant women’s experiences of routine obstetric abdominal ultrasound and showed that the examination was understood as more than a technical procedure for assessing fetal well-being. Across participants’ accounts, ultrasound was described as an experience in which emotional responses, visual perceptions, cognitive interpretations, communication, and privacy concerns were closely interconnected. Rather than eliciting a uniform emotional response, the examination was interpreted differently depending on women’s previous experiences, expectations, relationships with healthcare professionals, and the context in which care was provided.

### 4.1. The Emotionally Ambivalent Nature of Ultrasound

Participants’ accounts indicate that obstetric ultrasound may be experienced simultaneously as a source of anticipation and reassurance and as a moment of uncertainty. Women valued the opportunity to see the fetus and receive information about fetal development, while also remaining aware that the examination could identify an unexpected problem. This emotional coexistence was particularly evident among women with previous adverse obstetric experiences. Previous research similarly indicates that women who have experienced pregnancy loss may report heightened anxiety during subsequent pregnancies and increased sensitivity to information concerning fetal well-being [11].

The present findings are also consistent with studies suggesting that prenatal ultrasound may have a dual emotional meaning. Although visualization and information about fetal well-being may be reassuring, the examination can also make potential threats to fetal health more salient [1,7]. Similarly, previous research has described women’s efforts to evaluate fetal well-being and anticipate possible risks as an important component of the emotional experience of obstetric ultrasound [5]. Thus, reassurance should not necessarily be understood as the absence of anxiety. Rather, participants’ accounts suggest that reassurance and uncertainty may coexist within the same clinical encounter.

This interpretation is particularly relevant for women with previous adverse pregnancy experiences. For these women, the ultrasound appeared to carry meanings extending beyond routine clinical monitoring, as previous experiences could remain relevant to how the current examination was anticipated and interpreted. These findings suggest that the emotional experience of ultrasound is context-dependent and may be shaped by women’s personal histories rather than by the examination itself alone.

From a woman-centered care perspective, these findings support attention to the emotional context in which ultrasound is provided. Recognizing that women may approach the examination with both hope and apprehension may help healthcare professionals respond more sensitively to individual concerns, particularly when previous pregnancy loss or other adverse obstetric experiences are part of the woman’s history.

### 4.2. Visualizing the Fetus and the Embodiment of Motherhood

A central finding of this study was the importance participants attributed to seeing the fetus during ultrasound. Participants described visualization as making pregnancy more tangible and providing a concrete reference through which they could interpret fetal development and their own experiences of pregnancy. In this sense, the ultrasound image appeared to have meanings extending beyond its diagnostic function.

This finding is consistent with previous studies suggesting that fetal visualization may contribute to women’s perception of the fetus as a more concrete and individualized presence and may be associated with maternal–fetal attachment [8,12]. Previous research has particularly emphasized the potential emotional significance of three- and four-dimensional ultrasound in shaping perceptions of the fetus and experiences of motherhood [8]. The present findings extend this perspective by showing that even a routine abdominal ultrasound was described by participants as an emotionally meaningful visual encounter.

However, the findings should not be interpreted as demonstrating that ultrasound directly produces maternal attachment or changes women’s maternal identities. Rather, participants described the ultrasound image as a reference point through which they interpreted an experience already developing during pregnancy. For some women, seeing fetal growth and movement appeared to make an otherwise largely internal and bodily experience more concrete. For others, particularly women experiencing pregnancy for the first time, the novelty of the experience appeared to contribute to its emotional salience.

The relational meaning of visualization was also evident in participants’ descriptions of sharing ultrasound experiences with partners and close family members. Thus, the significance of the ultrasound image could extend beyond the clinical setting and become part of women’s interpersonal experiences of pregnancy. Within this context, fetal visualization may be understood as one element through which women gave personal and relational meaning to pregnancy rather than as an isolated technological effect.

### 4.3. Cognitive Meaning-Making and Active Engagement

The findings further indicate that women actively interpreted information obtained during an ultrasound rather than simply receiving it as clinical information. Participants connected fetal growth and development with bodily changes, emotional experiences, and their evolving understanding of pregnancy. This process is consistent with the concept of meaning-making, whereby individuals integrate new experiences into their existing understanding of themselves and their circumstances.

Previous research emphasized the role of prenatal care providers in shaping parents’ mental representations of the unborn child [17]. Consistent with this perspective, participants in the present study appeared to incorporate visual and informational aspects of ultrasound into their broader interpretation of fetal development and pregnancy. The examination, therefore, represented not only an opportunity to obtain information but also a context in which women interpreted its meaning for their own pregnancy.

Importantly, participants’ accounts suggest that the developing sense of motherhood was embedded within this broader interpretive process. Rather than viewing maternal identity as a direct outcome of ultrasound, the findings indicate that women connected what they saw on the screen with bodily sensations, fetal development, previous pregnancy experiences, and their expectations about motherhood. The meaning attributed to ultrasound, therefore, appeared to emerge through an ongoing process of interpretation rather than from the examination alone.

This interpretation also helps explain the close relationship between the emotional and cognitive dimensions identified in the study. The possibility of an unexpected finding could contribute to anxiety, while information about fetal development could provide reassurance. Similarly, seeing the fetus could provide a concrete reference through which women interpreted bodily and emotional changes. Ultrasound may therefore be understood as a meaning-making context in which women simultaneously interpreted fetal development, managed uncertainty, and reflected on their changing experience of pregnancy.

### 4.4. Communication as a Source of Psychological Reassurance

Communication emerged as an important relational dimension of participants’ ultrasound experiences. Women emphasized the value of clear explanations, opportunities to ask questions, eye contact, and responsive interactions with healthcare professionals. These accounts suggest that the meaning of ultrasound was shaped not only by what women saw or were told, but also by how the interaction surrounding the examination was experienced.

This finding is consistent with previous research, which highlighted the importance of communication during obstetric ultrasound in shaping parents’ emotional experiences [18]. Previous studies have also suggested that uncertainty, inadequate information, and prolonged silence may contribute to anxiety and emotional vulnerability during pregnancy [1,10]. The present findings are therefore broadly consistent with the view that communication forms an important part of the psychosocial experience of ultrasound.

Rather than interpreting communication as a direct cause of reduced anxiety, however, participants’ accounts suggest that women perceived supportive communication as contributing to feelings of reassurance, safety, and trust. Clear explanations appeared to help some participants interpret information about fetal health, while interpersonal responsiveness made the clinical encounter feel more manageable. Communication could therefore be understood as a form of relational support that acknowledged women’s uncertainty and emotional vulnerability.

This has implications for woman-centered prenatal care. Technical competence remains essential to ultrasound practice, but participants’ accounts indicate that the interpersonal context in which technical information is delivered also matters. Communication that is understandable, responsive, and sensitive to women’s questions may help create a clinical environment in which women feel more comfortable expressing concerns and interpreting information about their pregnancy.

### 4.5. Privacy, Gender, and Bodily Boundaries

The findings demonstrate that the ultrasound experience was also shaped by women’s perceptions of privacy, bodily exposure, and gender. Participants frequently described greater comfort with female healthcare professionals and emphasized the importance of limiting unnecessary exposure and maintaining an environment in which their bodily boundaries were respected. These findings are consistent with previous research conceptualizing obstetric ultrasound as an ethical, emotional, and relational encounter rather than solely a technical assessment [19,20].

The preference for female physicians expressed by many participants should, however, be interpreted within the study’s sociocultural and clinical context rather than as a universal preference among pregnant women. Expectations concerning modesty, gender interactions, bodily exposure, and appropriate interpersonal boundaries may influence how intimate healthcare encounters are experienced. Individual preferences and previous healthcare experiences may also shape women’s perceptions of comfort and safety.

The Turkish context is therefore relevant to interpreting these findings. In settings where cultural expectations concerning modesty and gender relations differ, women may attach different meanings to the gender of healthcare professionals or to bodily exposure during obstetric examinations. Similarly, the importance attributed to privacy may be influenced by the organization of healthcare services, the physical environment, previous experiences, and individual expectations. The findings should consequently be understood as contextually situated rather than culturally universal.

At the same time, the emphasis that participants placed on privacy is relevant beyond cultural differences. Regardless of individual preferences regarding the gender of healthcare professionals, respect for bodily autonomy and privacy represents an important dimension of person-centered care. Participants’ accounts indicate that environmental conditions, the presence of others, and the management of bodily exposure could influence how the examination was experienced emotionally. The contrasting report of mild physical discomfort further demonstrates that an examination could be emotionally acceptable while still involving some degree of physical discomfort.

Taken together, these findings suggest that the psychosocial experience of obstetric ultrasound is shaped by the interaction between technology, interpersonal care, and the physical environment. Privacy-sensitive practices, respectful communication, and attention to individual preferences may therefore be particularly relevant when providing woman-centered ultrasound care. At the same time, the culturally situated nature of participants’ accounts should be recognized, and comparative qualitative research across different healthcare and sociocultural settings may help clarify which aspects of these experiences are context-specific and which may be more widely shared.

### 4.6. Strengths and Limitations

This study provides an in-depth exploration of women’s experiences immediately following routine obstetric ultrasound and captures emotional, cognitive, relational, and embodied dimensions of the encounter. The concurrent conduct of data collection and reflexive analysis, the use of in-depth interviews, and the collaborative engagement of the research team supported the depth and transparency of the analysis.

Several limitations should be considered. First, the study was conducted in a single tertiary hospital and included only Turkish-speaking women, which may limit transferability to other healthcare and sociocultural contexts. Second, although maximum variation sampling was used to seek diversity in participant characteristics, the final sample was predominantly multiparous and in the third trimester. These characteristics should be considered when interpreting the findings.

Third, the interviewer was also the obstetrician who performed the ultrasound examination immediately before each interview and recruited participants. This dual clinical and research role may have introduced a power imbalance and social desirability bias, potentially influencing participants’ accounts of communication, reassurance, or clinical encounters. Although participants were informed that participation was voluntary and unrelated to their clinical care, such influence cannot be entirely ruled out.

Fourth, women were interviewed immediately after the ultrasound examination. Their accounts may therefore reflect immediate emotional responses and may not capture how perceptions of ultrasound develop or change over time. Longitudinal qualitative research could provide further insight into how the meanings attributed to ultrasound evolve throughout pregnancy.

Finally, women with severe psychiatric disorders or communication difficulties were excluded, and therefore, the findings may not reflect the experiences of all pregnant women, particularly those experiencing substantial psychological vulnerability. Although participants were informed of their right to decline to participate or withdraw from the interview, a formal, study-specific psychological referral pathway for interview-related distress was neither established nor documented in the original protocol. This limitation should be considered when interpreting the ethical safeguards of the study and highlights the importance of predefined distress-management and referral procedures in future qualitative research involving emotionally vulnerable pregnant women.

## 5. Conclusions and Implications

This study indicates that pregnant women may experience routine obstetric abdominal ultrasound as a multidimensional psychosocial encounter rather than solely as a diagnostic procedure. Participants described the examination in terms of reassurance and uncertainty, visual engagement with the fetus, cognitive interpretation of pregnancy, communication with healthcare professionals, and concerns regarding privacy and bodily exposure.

The findings highlight the importance of woman-centered care during obstetric ultrasound. Clear and responsive communication, attention to privacy, and sensitivity to individual preferences may help create a clinical environment in which women feel respected and emotionally supported. Attention may be warranted for women with previous pregnancy loss or other adverse obstetric experiences, for whom ultrasound may carry additional emotional significance.

Future research should examine women’s experiences across diverse sociocultural and healthcare settings, explore partners’ experiences, and investigate communication approaches during obstetric ultrasound. Longitudinal qualitative studies may also help clarify how women’s interpretations of ultrasound change across pregnancy.

In conclusion, obstetric ultrasound may be understood not only as a technology for assessing fetal well-being but also as a psychosocial encounter through which women interpret pregnancy, engage emotionally with the fetus, and reflect on their developing experience of motherhood. Recognizing these emotional, relational, cognitive, and embodied dimensions may contribute to more holistic and woman-centered prenatal care.

## Figures and Tables

**Table 1:** Sociodemographic and Obstetric Characteristics of Participants (n = 20).

Characteristic	Category	n	%
Age	24–29 years	7	35
	30–33 years	13	65
Pregnancy status	Primigravida	5	25
	Multiparous	15	75
Previous birth type	Previous cesarean birth	8	40
	Previous vaginal birth	7	35
Previous adverse obstetric history	Present	3	15
	Absent	17	85
Chronic medical condition	Present	7	35
	Absent	13	65
Gestational trimester	Second trimester	6	30
	Third trimester	14	70
Perception of ultrasound	Special/meaningful experience	18	90
	Routine medical procedure	2	10
Emotional response before ultrasound *	Excitement/curiosity	20	100
	Anxiety/fear accompanying excitement	15	75
Preference regarding physician gender	Female physician preferred	16	80
	No preference/not important	4	20
Information-seeking behavior before ultrasound	Internet/books/previous experience	14	70
	Limited/no preparation	6	30

Note. * Multiple responses were possible for emotional response categories. Percentages were calculated based on the total sample (n = 20). Percentages for previous birth type were calculated among participants with previous births (n= 15). Categorical information was obtained through direct questions included in the semi-structured interview guide and recorded systematically during the interviews.

## Data Availability

The datasets generated and/or analyzed during the current study are not publicly available due to confidentiality concerns, but are available from the corresponding author on reasonable request.

## Supplementary Materials

The following supporting information can be downloaded at https://www.mdpi.com/article/10.3390/healthcare14203472/s1. Supplementary File S1: COREQ Checklist. Consolidated Criteria for Reporting Qualitative Research (COREQ): 32-item checklist for interviews and focus groups.
