# Supplementary material for: Pregnant Women’s Experiences of Obstetric Abdominal Ultrasound: A Qualitative Study Exploring Emotional, Cognitive, and Relational Dimensions
Source: Healthcare (Basel). 2026 Oct 16;14(20):3472. doi: 10.3390/healthcare14203472 (PMC13704168; doi:10.3390/healthcare14203472)
Supplement: Supplementary file 1 [file healthcare-14-03472-s001.zip › healthcare-4551831-supplementary.pdf]

Supplementary File S1. COREQ Checklist

Consolidated Criteria for Reporting Qualitative Research (COREQ): 32-item checklist for interviews and focus groups. The locations below refer to the manuscript sections; page numbers may change after final formatting.

No.	COREQ item	Information reported in manuscript	Location / status
1	Interviewer/facilitator	Female obstetrician with experience in women's health and formal training in qualitative interviewing.	Methods – Research Team and Reflexivity
2	Credentials	Obstetrician.	Methods – Research Team and Reflexivity
3	Occupation	Obstetrician.	Methods – Research Team and Reflexivity
4	Gender	Female.	Methods – Research Team and Reflexivity
5	Experience and training	Experience in women's health and formal training in qualitative interviewing.	Methods – Research Team and Reflexivity
6	Relationship established	Some participants had previously encountered the researcher during routine clinical care; no ongoing therapeutic relationship existed at recruitment.	Methods – Research Team and Reflexivity
7	Participant knowledge of interviewer	Participants were informed about the study aims, voluntary participation, and their right to decline or withdraw without affecting care.	Methods – Research Team and Reflexivity
8	Interviewer characteristics/reflexivity	Reflexive field notes were maintained to document assumptions, clinical perspectives, and potential influences on interpretation; reflections were discussed within the research team.	Methods – Research Team and Reflexivity
9	Methodological orientation/theory	Qualitative descriptive design informed by constructivist and woman-centred perspectives.	Methods – Study Design
10	Sampling	Maximum variation purposive sampling.	Methods – Setting and Participants
11	Method of approach	Participants were recruited in the obstetric outpatient clinic; eligible women were approached/invited to participate.	Methods – Setting and Participants
12	Sample size	Twenty pregnant women participated.	Methods – Setting and Participants
13	Non-participation	The manuscript does not report the number of eligible women who declined	Not reported – verify before submission

		participation or reasons for non-participation.	
14	Setting of data collection	Individual interviews were conducted immediately after routine obstetric abdominal ultrasound in a private room within the outpatient clinic.	Methods – Data Collection
15	Presence of non-participants	Only the participant and interviewer were present during the interviews.	Methods – Data Collection
16	Description of sample	Participant characteristics are summarized in Table 1.	Results – Table 1
17	Interview guide	A semi-structured interview guide was developed from the literature and study aims and pilot-tested with two pregnant women; no changes were required after the pilot interviews.	Methods – Data Collection
18	Repeat interviews	No repeat interviews are reported; interviews were conducted as individual encounters immediately after the ultrasound examination.	Methods – Data Collection
19	Audio/visual recording	With written informed consent, all interviews were audio-recorded.	Methods – Data Collection
20	Field notes	Reflexive field notes documenting contextual observations and methodological reflections were maintained throughout data collection.	Methods – Data Collection / Research Team and Reflexivity
21	Duration	Interviews lasted between 30 and 45 minutes.	Methods – Data Collection
22	Data saturation / information power	Recruitment and data collection continued until sufficient information power was achieved; no new codes, themes, or conceptual insights emerged from successive interviews.	Methods – Setting and Participants
23	Transcripts returned	The manuscript does not state whether transcripts were returned to participants for comment or correction.	No. Transcripts were not returned to participants for comment or correction.
24	Number of data coders	The manuscript describes analysis by the researchers and regular team discussions, but the number of individual coders should be stated explicitly if applicable.	Two researchers (A.S.B. and M.A.A.) coded the transcripts.
25	Description of coding tree	Initial inductive codes were generated; codes were organized into potential themes and subthemes, which were developed and refined through continuous comparison across the dataset.	Methods – Data Analysis

26	Derivation of themes	Themes were developed through reflexive thematic analysis, with interpretation informed by the interview guide and literature as sensitizing concepts.	Methods – Data Analysis
27	Software	The manuscript does not specify whether qualitative data analysis software was used.	Two researchers (A.S.B. and M.A.A.) coded the transcripts.
28	Participant checking	The manuscript should explicitly state whether participant/member checking was undertaken.	No. Participant/member checking was not undertaken.
29	Quotations presented	Participant quotations are presented in the Results using participant identifiers.	Results
30	Consistency between data and findings	Themes and interpretations are supported by participant accounts and illustrative quotations.	Results
31	Clarity of major themes	The overarching themes are clearly identified and described.	Results – Thematic findings
32	Clarity of minor themes	Subthemes/interrelated dimensions are described within the thematic structure.	Results – Thematic findings
